# Assessing fear of childbirth in Malaysia: development and psychometric testing of the Malaysian Fear of Birth questionnaire (MyFOB)

**DOI:** 10.3389/fpubh.2025.1714125

**Published:** 2026-01-09

**Authors:** Aida Kalok, Ixora Kamisan Atan, Shalisah Sharip, Shamsul Azhar Shah

**Affiliations:** 1 Department of Public Health Medicine, Faculty of Medicine, Universiti Kebangsaan Malaysia, Kuala Lumpur, Malaysia; 2 Department of Obstetrics and Gynaecology, Faculty of Medicine, Universiti Kebangsaan Malaysia, Kuala Lumpur, Malaysia; 3 Department of Psychiatry, Faculty of Medicine, Universiti Kebangsaan Malaysia, Kuala Lumpur, Malaysia

**Keywords:** Asia, childbirth, fear, Malaysia, pregnancy, tokophobia

## Abstract

**Introduction:**

Fear of childbirth or tokophobia refers to the pathological fear of childbirth, which affects the daily life of pregnant women. Childbirth fear is multidimensional and differs across cultures. We aimed to develop and validate the first Malaysian Fear of Birth questionnaire (MyFOB) among the multiethnic Malaysian women.

**Methods:**

The research was conducted in the antenatal clinic, Hospital Canselor Tuanku Muhriz, Kuala Lumpur. A mixed-methods, exploratory sequential approach was applied. Phase 1 involved a qualitative exploration of childbirth fear through one-to-one interviews, while phase 2 comprised questionnaire development based on the qualitative results, literature review and experts’ input. Psychometric properties of the questionnaire were assessed through exploratory factor analysis (EFA), face validation, and reliability testing. Subsequently, confirmatory factor analysis (CFA) was performed to determine the model fit of the final version of MyFOB. EFA and CFA were conducted on two separate cohorts.

**Results:**

Twelve qualitative interviews yielded five themes. Subsequently, one hundred thirty-seven items were generated and underwent content validation by five experts. The construct validity of the initial 43-item questionnaire was assessed through EFA. Phase 2 cohorts for EFA and CFA were 257 and 341, respectively. Principal component analysis resulted in MyFOB, which comprised 20 items accounting for 65.6% of the total variance. These items were grouped into four factors: 1) Labour & delivery, 2) Pain, 3) Maternal emotion, and 4) Healthcare professionals. The pilot study on sixty-one women revealed good reliability (Cronbach *α* =0.88) while confirmatory factor analysis demonstrated a good model fit.

**Discussion:**

The Malaysian Fear of Childbirth Questionnaire (MyFOB) is a reliable and valid tool that can be utilised in routine antenatal care to identify vulnerable women.

## 1 Introduction

Fear of childbirth (FOC) or tokophobia is a pathological fear of childbirth that affects a pregnant woman’s daily life and well-being (1). The lack of international consensus on standard diagnostic criteria and variable assessment tools contributed to the various prevalences between regions (2). The pooled prevalence of tokophobia worldwide was 14% (3), with a recent review demonstrating greater prevalences among Asians (range 56.6–84.4%) (4). Variations in the developing countries’ social, political, and health systems, compared to those of their developed Western counterparts, may contribute to the difference (5).

Factors such as maternal age, education level, marital status, and socio-economic status have a significant influence on childbirth fear. Greater levels of tokophobia are associated with nulliparity, unplanned pregnancy, infertility and previous miscarriage (4). Maternal depression and anxiety are among the positive predictors of tokophobia, while spousal and social support, and a well-functioning family, protect expectant mothers against childbirth fear (6).

Tokophobia adversely impacts women during and after childbirth. FOC is linked to a longer labour, increased use of epidural, and a greater risk of emergency caesarean section (7). A hormonal stress response through elevated plasma adrenaline, leading to ineffective uterine contractions, may explain the prolonged labour duration (8). Women with tokophobia are also more likely to report negative birth experiences and postpartum symptoms of depression and anxiety (9, 10), resulting in dysfunctional mother-baby bonding. Data from China demonstrated that a high level of FOC is associated with a 67% reduction in exclusive breastfeeding (9). Negative birth experience also adversely influences women’s attitude towards subsequent delivery, leading to an increase in maternal desire for elective caesarean section (11).

Childbirth fear is multidimensional as it involves various fears and anxieties related to the physical, emotional, psychological, and social aspects of pregnancy and delivery. Qualitative research revealed various contents of tokophobia that include fear of pain, worry about labour unpredictability, loss of self-control, feeling of loneliness, and risk of birth complications (12). Factors characterising a population, such as ethnicity, religion, social structures, and social norms, will also influence maternal childbirth fear (13), making it paramount to understand tokophobia in the local context (12).

Standardised FOC assessment tool may be restrictive considering that expectant mothers are from various cultural backgrounds, with differing perceptions and beliefs regarding birth (14). Current literature indicates that most FOC questionnaires originated from Western countries (15). A recent review found that the most utilised diagnostic tools among Asian cohorts were the Wijma Delivery Expectancy Questionnaire Part A (WDEQ-A) and the Childbirth Attitude Questionnaire (CAQ) (6), which were developed based on Swedish and American populations, respectively (16, 17). Despite being validated in various countries, doubt persists about the translated versions’ validity due to cultural nuances (14, 18), as the instruments may exhibit different psychometric properties in other languages and cultural contexts (19).

Our country, Malaysia, has a multicultural society, with three main ethnic groups, Malay, Chinese and Indian. Published data on childbirth fear among Malaysians remains lacking, and no validated Malaysian instrument exists to measure tokophobia. We aimed to explore the fear of childbirth among our women and produce a locally adapted diagnostic tool.

## 2 Materials and methods

Our research was conducted at the antenatal clinic, Hospital Canselor Tuanku Muhriz (HCTM), Kuala Lumpur, Malaysia. HCTM is a tertiary referral centre and a teaching hospital for the National University of Malaysia. Prior ethical approval was obtained from the Universiti Kebangsaan Malaysia (UKM) Research Ethics Committee (Reference: FF-2023-316).

**Figure 1:**
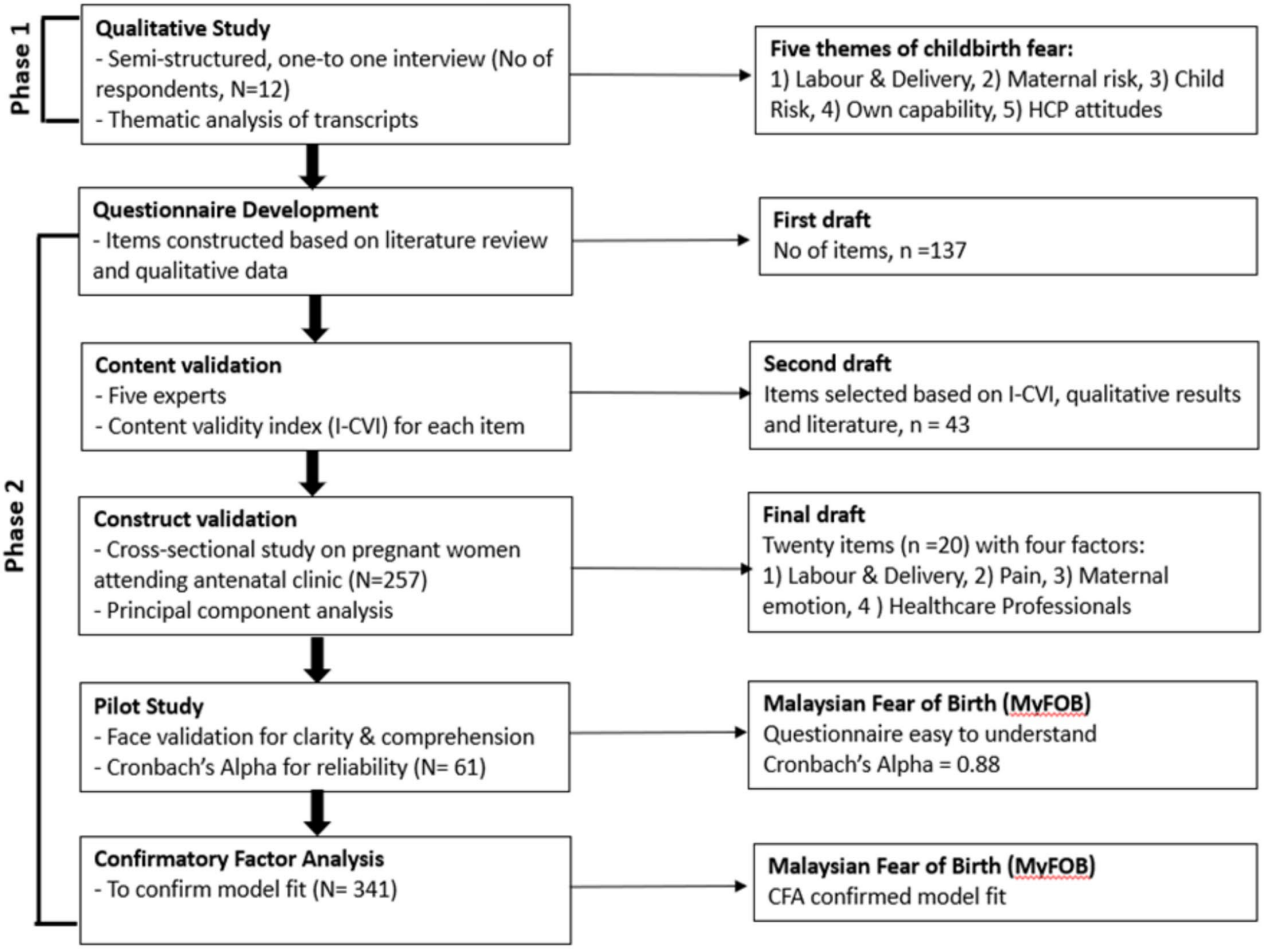
Malaysian Fear of Birth (MyFOB) development process.

A mixed-methods approach in the form of an exploratory sequential study was applied. Phase 1 involved a qualitative study exploring childbirth fear among Malaysian women. Phase 2 consisted of questionnaire development and psychometric validation, including exploratory factor analysis (EFA) and confirmatory factor analysis (CFA). EFA and CFA were conducted on two separate cohorts. The following describes our study timeline: Phase 1, January–June 2024; Phase 2 EFA, January–March 2025; and Phase 2 CFA, March–May 2025. Figure 1 illustrates the questionnaire development process.

### 2.1 Phase 1: Qualitative study

Eligible pregnant women who attended their follow-ups in the antenatal clinic were approached for the qualitative study. Inclusion criteria were aged 18 and above, and could converse in Malay or English. Women who were diagnosed with a miscarriage or fetal anomaly were excluded.

Participants were recruited through purposive sampling to ensure diversity and representation across key demographic and obstetric characteristics such as ethnicity, parity and previous mode of delivery. Consented women underwent face-to-face, semi-structured interviews in a closed consultation room to ensure confidentiality.

The interview guide consists of questions that explore: (1) women’s perceptions and expectations of the childbirth process, (2) support during labour, and (3) healthcare provision. The interviews were recorded, transcribed verbatim and thematically analysed. Patient recruitment was stopped once data saturation was achieved. To ensure data reliability, the transcripts coding was performed by three independent researchers.

### 2.2 Phase 2: Questionnaire development and validation (quantitative)

The initial draft of the questionnaire was developed based on the qualitative data and literature review. The first version was subjected to content validation by five experts who provided different professional perspectives (an epidemiologist, a clinical psychologist, an obstetrician and two midwives). Each assessor was asked to score each item using a Likert scale from 1 to 4 for its relevance. The experts were also encouraged to provide constructive comments to improve individual items. The item content validity index (I-CVI) was then calculated. The selection of items for the subsequent questionnaire draft was based on the I-CVI scoring, expert feedback, qualitative results, and current literature.

The construct validity of the second version was assessed through a cross-sectional study that used the same inclusion and exclusion criteria as those applied in the qualitative research. The sample size was based on a ratio of five respondents per item (20). Participant recruitment was based on convenience sampling. Eligible women who attended the antenatal clinic were invited to complete a self-administered electronic questionnaire (Google Form), with a consent section included. Participants were required to scan a QR code provided by the researcher to access the electronic form. Individuals without a mobile phone or internet access were supplied with a tablet computer to answer the questionnaire. Each questionnaire item consists of a statement with a Likert scale response from 1 (strongly disagree) to 7 (strongly agree). Socio-demographic and clinical data were also collected.

The pilot study was conducted to assess the face validity and internal consistency of the final version of our questionnaire. The sample size required was 30 (21).

We conducted confirmatory factor analysis on the validated questionnaire. A Google form of the assessment tool was distributed to a separate cohort between March and May 2025, with a minimum sample size requirement of 200 (20). Similarly, convenience sampling was applied to recruit respondents among the antenatal clinic attendees.

### 2.3 Statistical methods

#### 2.3.1 Phase 1

Thematic analysis for the qualitative data was performed using the ATLAS.ti 25 Windows software. Krippendorff’s Cu-*α* was calculated to determine intercoder reliability, and a value >0.8 indicates a satisfactory level of agreement between coders (22).

#### 2.3.2 Phase 2

For content validation index calculation, the expert’s rating was subsequently recoded (1 for relevance scale of 3 or 4, and 0 for relevance scale of 1 or 2). The item content validity index (I-CVI) was calculated from the proportion of experts who rated the item as relevant over the total number of evaluators. An I-CVI value of 0.78 or higher indicates good content validity (23).

We conducted the exploratory factor analysis using the Statistical Package for Social Sciences (SPSS) version 26.0. The sampling adequacy was evaluated through the Bartlett’s test of sphericity and the Kaiser–Meyer–Olkin (KMO) test. Kaiser rule (Eigenvalue>1.0), scree plot and parallel analysis were applied to determine the number of dimensions to extract (24). Items with a minimum factor loading of 0.4 were retained (25). Principal component analysis was used to reduce items and arrange them into the relevant domains. The refined questionnaire was subjected to reliability testing in the subsequent pilot study. Cronbach’s Alpha, with an acceptable cut-off of 0.70, was used to assess the questionnaire’s internal consistency (25).

Confirmatory factor analysis was performed using the AMOS software. The model fit was assessed by the following indices and their respective thresholds: ꭓ^2^/df < 3; adjusted goodness of fit index (AGFI) > 0.85; goodness of fit index (GFI) > 0.85; comparative fit index (CFI) > 0.90; incremental fit index (IFI) > 0.90; root mean square error of approximation (RMSEA) < 0.08, and Tucker-Lewis index (TLI) ≥ 0.90 (25, 26).

## 3 Results

### 3.1 Phase 1

Twelve women were interviewed. The initial transcript assessment identified 519 quotations. Through deductive analysis and collaborative discussion, 45 codes were developed, encompassing 126 quotations. Krippendorff’s Cu-*α* of 0.828 was suggestive of good intercoder reliability.

**Figure 2:**
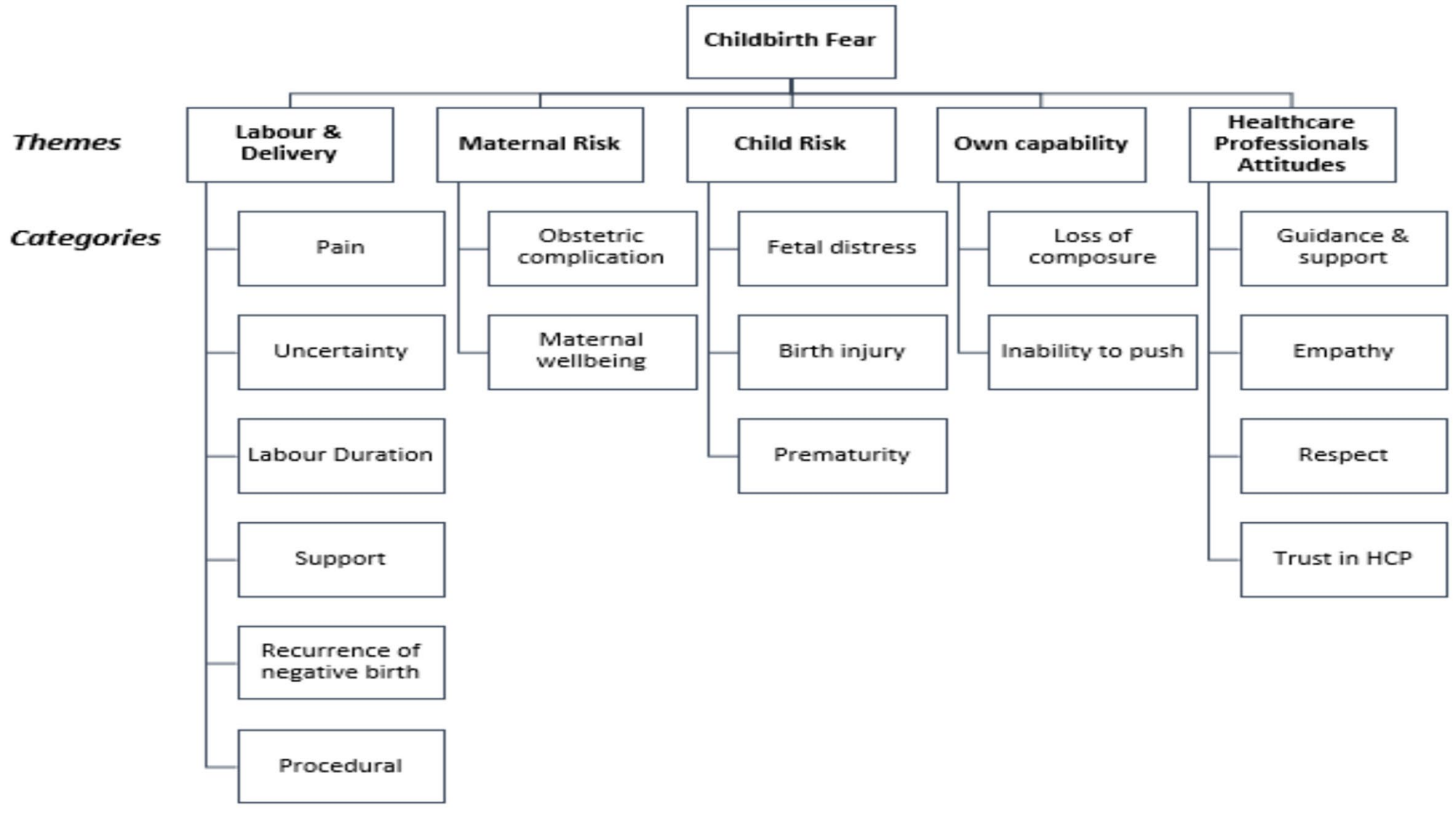
Themes and categories of childbirth fear among Malaysian women.

Five themes emerged from the data analysis: (1) Labour and Delivery, (2) Maternal Risk, (3) Child Risk, (4) Own Capability, and (5) Healthcare Professionals’ (HCP) Attitude. Fear of labour and delivery included that of pain, clinical procedures, prolonged labour, absence of support and unpredictable labour course. Maternal risk highlighted the women’s concern about potential obstetric complications as well as their own well-being. Fetal distress, birth injury and prematurity made up the risk to the unborn child. Inability to push and losing composure were among the women’s worries about their capabilities. Finally, the theme of HCPs’ attitudes discussed the guidance, support, and empathy shown by the healthcare providers. Figure 2 summarises the theme and categories of childbirth fear among Malaysian women.

### 3.2 Phase 2

The first draft of the questionnaire consisted of 137 items generated from the results of the qualitative study and a literature review. I-CVI scores ranged between 0.20 and 1.00. Thirty-one items were chosen based on I-CVI (≥0.8), while twelve were selected based on the previous qualitative results and literature review. The construct validity of the resultant 43-item questionnaire was assessed in the subsequent cross-sectional study using EFA. CFA was conducted on a separate cohort to determine the model fit of MyFOB. The sample sizes for the EFA and CFA were 257 and 341, respectively.

The majority of our respondents were less than 35 years old (70.9%), with a median age (IQR) of 32 (7). Most participants were Malays (86.0%), received tertiary education (79.6%), and were in full-time employment (72.7%). Primigravidas made up just over a third of our respondents (35.3%), and approximately 25% of women had a previous miscarriage. The median gestation (IQR) was 32 (11) weeks.

Table 1 shows the demographic and clinical characteristics of both the EFA and CFA groups. Chi-square test confirmed no significant differences between the two.

**Table 1:** Maternal characteristics for development and confirmation samples.

Maternal characteristics	Development (PCA sample)	Confirmation (CFA sample)	Pearson chi square
	*n* = 257	*n* = 341	*p* value
Age (years), median (IQR)	31 (7)	32 (6)	*p* = 0.75
18–34	184 (71.6)	240 (70.4)	
35 and above	73 (28.4)	101 (29.6)	
Ethnicity			
Malay	219 (85.2)	295 (86.5)	*p* = 0.66
Chinese	17 (6.6)	23 (6.7)	
Indian	9 (3.5)	10 (2.9)	
Bumiputera	11 (4.3)	9 (2.6)	
Others	1 (0.4)	4 (1.2)	
Malay	219 (85.2)	295 (86.5)	*p* = 0.65
Non-Malay	38 (14.8)	46 (13.5)	
Education			
Lower	56 (21.8)	66 (19.4)	*p* = 0.46
Higher	201 (78.2)	275 (80.6)	
Employment			
Employed	192 (74.7)	262 (76.8)	*p* = 0.55
Housewife	65 (25.3)	79 (23.2)	
Household income (RM), median (IQR)	5,000 (4,000)	5,000 (4,800)	*p* = 0.90
<4,999 B40	108 (42.0)	141 (41.3)	
RM 5000–9,999 M40	122 (47.5)	160 (46.9)	
>RM10000 T20	27 (10.5)	40 (11.7)	
First Pregnancy	87 (33.9)	124 (36.4)	*p* = 0.53
Parity, median (IQR)	1 (2)	1 (2)	*p* = 0.70
Nulliparous	91 (35.4)	126 (37.0)	
Multipara	166 (64.6)	215 (63.0)	
Previous miscarriage	64 (24.9)	84 (24.6)	*p* = 0.94
Gestation (weeks), median (IQR)	32 (11)	32 (8)	*p* = 0.50
First trimester (up to 13 weeks)	13 (5.1)	13 (3.8)	
Second trimester (14–27 weeks)	64 (24.9)	75 (22.0)	
Third trimester (28–40 weeks)	180 (70.0)	253 (74.2)	
Early (1^st^ & 2^nd^ trimester)	77 (30.0)	88 (25.8)	*p* = 0.26
Late (3^rd^ trimester)	180 (70.0)	253 (74.2)	
Medical condition, *n* (%)			
Medical condition	93 (36.2)	126 (37.0)	*p* = 0.85
No medical condition	164 (63.8)	215 (63.0)	

#### 3.2.1 Principal component analysis and reliability

**Figure 3:**
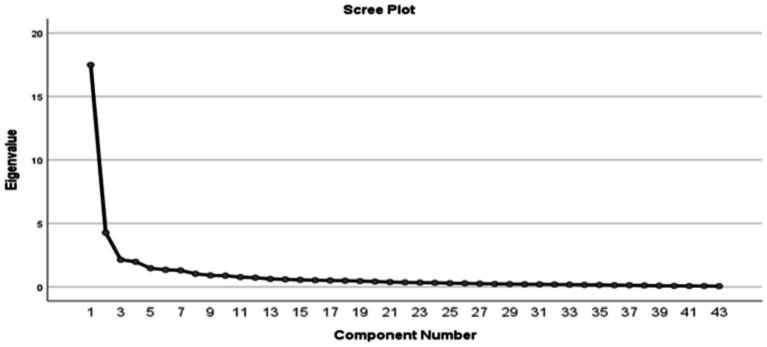
The scree plot for the initial exploratory factor analysis.

Adequate sampling was confirmed by the KMO value of 0.925 and significant Bartlett’s test (chi-square = 9610.574, *p* < 0.001). The exploratory factor analysis of the 43 initial items was conducted using oblique rotation (Promax). The results revealed eight components explaining 72.14% of the total variance. We retained four components based on the scree plot inspection (Figure 3) and the results of parallel analysis that showed only four components with eigenvalues exceeding the corresponding criterion values for a randomly generated data matrix of the same size. The basis for this decision was that the four factors represented our dataset’s most significant variance or information.

We conducted the second round of principal component analysis using a four-factor solution and performed item reduction based on the communalities and the correlation matrix. The resultant questionnaire consisted of twenty items that account for 65.5% of the total variance and were categorised into four domains: (1) Labour & Delivery, (2) Pain, (3) Maternal Emotion, (4) Healthcare Professionals (Table 2). Face validation during the pilot study confirmed the clarity and understandability of the scale. The overall Cronbach’s Alpha was 0.88, with values for each domain ranging between 0.72 and 0.91. Table 3 demonstrates the factor loading of each item and the Cronbach’s Alpha values.

**Table 2:** Individual items and domains of the Malaysian Fear of Birth (MyFOB) scale.

Item no	Domain	Question
1*	E	Saya berasa yakin untuk bersalin (I feel confident about giving birth)
2	P	Saya bimbang tentang sakit semasa bersalin (I worry about labour pain)
3	P	Saya bimbang tentang pemeriksaan faraj (vagina) sepanjang proses bersalin (I worry about having vaginal examinations during labour)
4	P	Saya bimbang tidak mendapat ubat tahan sakit yang berkesan semasa sakit bersalin (I am worried about not getting effective pain relief)
5	P	Saya risau tentang jangkamasa proses bersalin yang lama (I worry about a long labour)
6	P	Saya bimbang suami saya tidak dapat menemani saya sewaktu bersalin (I am worried that my husband will not be able to accompany me during labour)
7*	E	Saya akan berasa tenang semasa bersalin (I will feel calm during labour)
8	LD	Saya takut ditinggalkan bersendirian semasa proses bersalin (I have fear of being left along during labour)
9*	HP	Saya yakin kakitangan kesihatan akan melayan saya dengan baik (I am confident the healthcare staff will be kind to me)
10*	E	Saya berasa teruja untuk bersalin (I feel excited about giving birth)
11	LD	Saya bimbang tidak dapat meneran dengan betul semasa bersalin (I worry about not pushing correctly during childbirth)
12*	HP	Saya yakin doktor & jururawat akan membimbing saya semasa bersalin (I’m confident staff will guide me)
13	LD	Saya bimbang akan hilang kawalan diri semasa bersalin (I worry I will lose control of myself during labour)
14	LD	Saya bimbang dimarahi oleh petugas kesihatan (I worry about getting scolded by the healthcare staff)
15	P	Saya takut mengalami episiotomi (potongan di bahagian faraj) (I fear of having episiotomy/ being cut in the vagina)
16	LD	Saya risau mengalami komplikasi sewaktu bersalin (I worry that my labour will be complicated)
17	LD	Saya bimbang bayi saya akan lemas semasa proses kelahiran (I worry that my baby will feel distressed during childbirth)
18	LD	Saya bimbang saya perlu bersalin melalui pembedahan (I worry that I may need caesarean delivery)
19*	HP	Saya percaya bahawa petugas kesihatan yang menjaga saya akan membuat keputusan yang tepat untuk saya (I trust that the healthcare staff will make the right decision for me)
20	LD	Saya bimbang tentang pemulihan badan saya selepas bersalin (I worry about my recovery from childbirth)

* Reverse scoring; E, maternal emotion; HP, health professionals; LD, labour & delivery; P, pain.

**Table 3:** Item factor loading & Cronbach’s Alpha values.

Item position	MyFOB item	Components			
		LD	*P*	E	HP
13	I worry I will lose control of myself during labour	0.907			
18	I worry that I may need caesarean delivery	0.858			
14	I worry about getting scolded by the healthcare staff	0.754			
20	I worry about my recovery from childbirth	0.750			
11	I worry about not pushing correctly during childbirth	0.740			
16	I worry that my labour will be complicated	0.695			
17	I worry that my baby will feel distressed during childbirth	0.548			
8	I have fear of being left alone during labour	0.524			
4	I am worried about not getting effective pain relief		0.954		
3	I worry about having vaginal examinations during labour		0.684		
6	I am worried that my husband will not be able to accompany me during labour		0.678		
2	I worry about labour pain		0.660		
15	I fear of having an episiotomy or being cut in the vagina		0.527		
5	I worry about a long labour		0.522		
1*	I feel confident about giving birth (REV)			0.872	
10*	I feel excited about giving birth (REV)			0.867	
7*	I feel calm during labour (REV)			0.808	
9*	I am confident the HCP will be kind to me (REV)				0.888
19*	I trust HCP will make the right decision (REV)				0.839
12*	I am confident staff will guide me (REV)				0.826
Cronbach’s alpha (overall = 0.88)		0.86	0.88	0.72	0.91

* Reverse scoring; E, maternal emotion; HP, health professionals; LD, labour & delivery; P, pain.

#### 3.2.2 Confirmatory factor analysis

**Figure 4:**
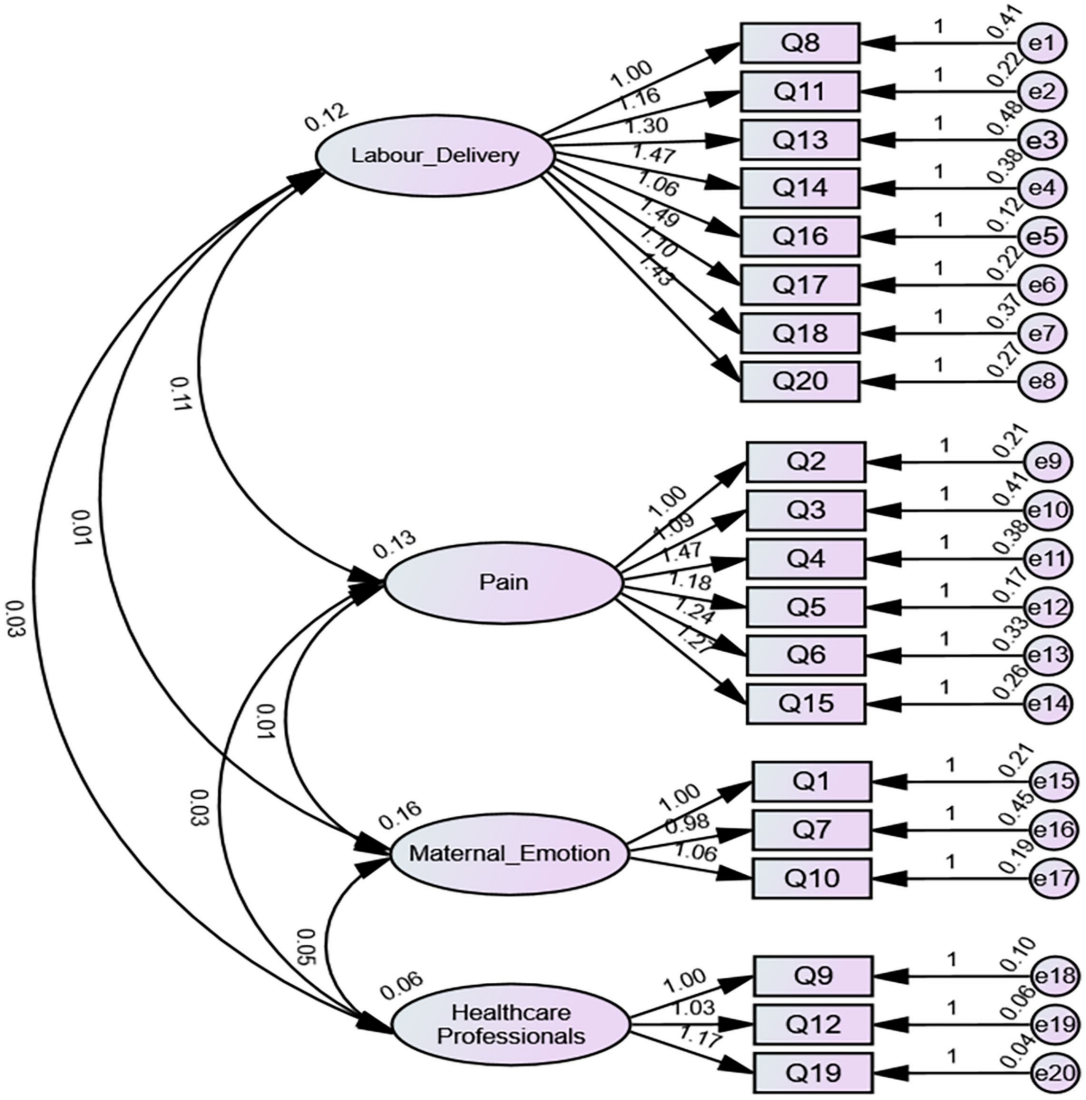
Confirmatory factor analysis of the MyFOB four-factor model.

The 20-item questionnaire was subjected to confirmatory factor analysis using a separate cohort from the questionnaire development phase. The results confirmed model fit (Table 4). Figure 4 depicts the final structure of MyFOB in CFA.

**Table 4:** Fit model indices of MyFOB.

Model	ꭓ^2^/df	GFI	AGFI	CFI	IFI	TLI	RMSEA
Acceptable value	<3.000	>0.850	>0.850	>0.900	>0.900	>0.900	<0.080
MyFOB: 4-factor model	2.287	0.902	0.874	0.906	0.907	0.891	0.062

## 4 Discussion

Our study is the first to explore fear of childbirth among Malaysian women and develop a population-based diagnostic tool for the condition. MyFOB demonstrated a four-factor solution encompassing concerns about the delivery process, pain, caregiver interaction, and maternal emotions. WDEQ-A was developed as a unidimensional instrument; however, studies in the UK and Australia identified four domains: fear, lack of positive anticipation, isolation, and riskiness (27, 28). Similarly, while the CAQ demonstrated single-factor structure among Thai and Greek women (29, 30), the Chinese version revealed four dimensions of fear related to labour pain, baby safety, loss of control and the birthing environment (31).

Exploratory factor analysis identified pain as a significant component of MyFOB. The fear of labour pain is one of the most prevalent concerns among expectant mothers (32, 33), especially the nulliparous (34, 35). Pain related to episiotomy and vaginal tear also contributes to FOC among Malaysians, in line with other published studies (12, 36).

Concern about inadequate labour analgesia has the highest loading in the pain subdomains, indicating the importance of pain relief during childbirth in our cohort. Interestingly, Japanese women reported opposing views on obstetric analgesia as they believed that sharing pain and suffering with their baby during labour was essential for a satisfying birth and would make them confident mothers (12).

Although vaginal examination is a vital part of intrapartum care and tolerated by labouring women, the experience can be painful, distressing and even embarrassing (37). Unsurprisingly, Malaysian women are concerned about a long labour, during which they have to face not only painful contractions but also repeated vaginal assessments. Spousal support in labour is vital to our women and is perceived to have a protective effect against pain. Partner’s supportive involvement during childbirth has been observed to lessen maternal pain and anxiety, shorten labour and reduce analgesia use (38). Pain perception in labouring women is heightened by fear and anxiety, which can be reduced through spousal emotional support in the form of encouragement, reassurance and continuous presence (39, 40).

The Labour & Delivery component of MyFOB include (1) risks to mother and fetus, and (2) maternal capacity and performance during childbirth. Expectant mothers perceived birth as a dangerous event, and fear of personal harm or injury as a result of childbirth has been consistent throughout the literature (32, 33). Women worry about their safety during labour and delivery, including the risk of having an obstetric complication such as haemorrhage, requiring emergency caesarean section and dying during childbirth (19, 41), in line with our findings.

Women often expressed greater concern for their infants’ well-being than their own, as they considered themselves responsible for their babies’ health (35). Therefore, putting their unborn children’s health at risk during delivery has become one of the common fears among expectant mothers (32). Apart from suffering from physical injury or harm during birth (33), women are also frightened that their babies might be delivered prematurely, born deformed, suffocated, or even die at birth (32, 35). Lebni et al. found that Iranian women reported concern about the umbilical cord wrapped around the baby’s neck (35), similar to our women who were worried about cord entanglement that may cause fetal distress.

Consistent with previous studies, our subjects expressed fear of losing control and being unable to perform during childbirth (33). Women were afraid of losing control over their bodies and displaying embarrassing behaviour, such as screaming and crying while giving birth (19, 41, 42). Expectant mothers also worry about their physical ability to deliver vaginally and reported concerns such as not having adequate energy to push or not pushing correctly (43). The possibilities of a big baby, a narrow pelvis and insufficient uterine contractions also contributed to maternal concerns about their capacity to achieve a normal birth (33, 43).

Feedback and support from clinicians strongly influenced Malaysians’ perception of how they would perform in labour and delivery, as evidenced by the relevant factor loadings. Interactions with healthcare providers can be a source of fear for labouring women. Apart from not receiving enough support during childbirth (33), women also expressed significant concern about inadequate medical care due to a lack of professional competence (44). Our women reported fear of abandonment by their clinicians, in line with the findings of studies on Western populations (19, 36). Malaysians were also worried about being scolded by their caregivers during childbirth. Similarly, Sercekus et al. found that Turkish women were afraid of being yelled at or making an error during labour, with some believing that they would not receive enough support and would be left alone at birth (41).

The attitudes of healthcare providers during labour are important to our cohort. Malaysian women valued the guidance and kindness of the health professionals and were confident they would make the right clinical decision. Guidance and information from midwives were shown to reduce childbirth fear as they helped women become more involved in the birthing process (45). A caring and supportive relationship between expectant mothers and their midwives protects against tokophobia, as women’s self-esteem increases when they feel listened to and respected (46). Women also realised that they had no control over the circumstances during labour and therefore should trust their caregivers and rely on their expertise (45). The unpredictability of childbirth contributes to women’s fear, and allowing skilled professionals to make decisions for them may be perceived as a relief to some women, as the responsibility is not theirs anymore (47).

We include the assessment of maternal emotion as part of our diagnostic tool. The subdomain evaluates women’s confidence and how calm they perceive themselves during labour, which reflects self-efficacy. Maternal self-efficacy is a dynamic cognitive process that enables expectant mothers to assess their ability to respond appropriately to various labour circumstances (6). Self-efficacy plays a mediating role in the relationship between maternal resilience and childbirth fear (48). Women with high stress resilience utilise most of their psychological resources to strengthen their ability to handle childbirth, resulting in greater confidence to face labour and subsequently reducing tokophobia (48). Increased mental resilience is also associated with higher levels of cognitive and psychological well-being (6), instilling a sense of calmness in expectant mothers. Women often described the feeling of excitement, happiness, and calm during early labour (49), reflecting their self-confidence. Women’s favourable perceptions of their coping styles and confidence in their capacity to go through labour are crucial, as they contribute to mothers’ positive childbirth experience (49).

### 4.1 Strengths and limitations

MyFOB is the first FOC assessment tool developed based on qualitative exploration of childbirth fear among our population, making it culturally appropriate for Malaysians. The validation of its psychometric properties and model fit on two cohorts confirms its strength as a diagnostic scale. MyFOB addresses the multidimensional nature of tokophobia, integrating subdomains that reflect concern represented in both the WDEQ-A and CAQ.

Our twenty-item questionnaire is shorter and more convenient to answer than the commonly utilised WDEQ-A, allowing a wider clinical use beyond research settings. MyFOB may also be used in other populations in the surrounding Southeast Asia region due to the similarity in culture and beliefs. Further instrument validation is required to determine its cross-cultural applicability.

Our research was conducted in a tertiary hospital in Kuala Lumpur. Most of our respondents had higher education and socio-economic status than the rest of the country. Our research, therefore, might be missing input from those from a lesser background. Further study using MyFOB should include more diverse groups of women from other states in Malaysia, including those in rural areas. This study’s use of a self-administered questionnaire may also subject our data to potential recall and response biases.

## 5 Conclusion

Our pioneer research into fear of childbirth in Malaysia found similar maternal concerns to those of global populations. The validated MyFOB covers all the significant themes of fear relevant to our cohort. Malaysian women worry about pain, risk of harm to themselves and their babies, and how they perform in labour. Healthcare professionals’ attitudes have been shown to influence women’s perception of their capacity to achieve a normal delivery. Finally, MyFOB’s emotion domain has allowed us to assess maternal self-efficacy and confidence in childbirth.

Future research should further evaluate the MyFOB’s predictive validity and utility in clinical settings to identify women needing psychological support. Early recognition of childbirth fear is pivotal for implementing timely evidence-based interventions that reduce the burden of tokophobia and promote a positive childbirth experience among expectant mothers.

## Data Availability

The original contributions presented in the study are included in the article/supplementary material, further inquiries can be directed to the corresponding author.

## References

[1] Demšar K Svetina M Verdenik I Tul N Blickstein I Velikonja VG. Tokophobia (fear of childbirth): prevalence and risk factors. J Perinat Med. (2018) 46:151–4. doi: 10.1515/jpm-2016-0282, 28379837

[2] Nilsson C Hessman E Sjöblom H Dencker A Jangsten E Mollberg M . Definitions, measurements and prevalence of fear of childbirth: a systematic review. BMC Pregnancy Childbirth. (2018) 18:28. doi: 10.1186/s12884-018-1659-7, 29329526 PMC5766978

[3] O'Connell MA Leahy-Warren P Khashan AS Kenny LC O'Neill SM. Worldwide prevalence of Tocophobia in pregnant women: systematic review and Meta-analysis. Acta Obstet Gynecol Scand. (2017) 96:907–20. doi: 10.1111/aogs.13138, 28369672

[4] Kalok A Kamisan Atan I Sharip S Safian N Shah SA. Factors influencing childbirth fear among Asian women: a scoping review. Front Public Health. (2024) 12:1448940. doi: 10.3389/fpubh.2024.1448940, 39877914 PMC11772208

[5] Sanjari S Chaman R Salehin S Goli S Keramat A. Update on the global prevalence of severe fear of childbirth in low-risk pregnant women: a systematic review and meta-analysis. Int J Womens Health Reprod Sci. (2022) 10:3–10. doi: 10.15296/ijwhr.2022.02

[6] Kalok A Kamisan Atan I Sharip S Safian N Shah SA. Psychosocial determinants of childbirth fear among Asian women: a scoping review. Healthcare (Basel). (2025) 13:1535. doi: 10.3390/healthcare13131535, 40648560 PMC12248780

[7] Zhou XL Liu H Li XH Li F Zhang SM Zhang SR. Mediating effects of social support between antenatal depression and fear of childbirth among nulliparous woman. Ann Palliat Med. (2021) 10:6399–409. doi: 10.21037/apm-21-854, 34237961

[8] Sydsjö G Angerbjörn L Palmquist S Bladh M Sydsjö A Josefsson A. Secondary fear of childbirth prolongs the time to subsequent delivery. Acta Obstet Gynecol Scand. (2013) 92:210–4. doi: 10.1111/aogs.12034, 23066797

[9] Yin A Shi Y Heinonen S Räisänen S Fang W Jiang H . The impact of fear of childbirth on mode of delivery, postpartum mental health and breastfeeding: a prospective cohort study in Shanghai, China. J Affect Disord. (2024) 347:183–91. doi: 10.1016/j.jad.2023.11.054, 38007102

[10] Rúger-Navarrete A Vázquez-Lara JM Antúnez-Calvente I Rodríguez-Díaz L Riesco-González FJ Palomo-Gómez R . Antenatal fear of childbirth as a risk factor for a bad childbirth experience. Healthcare. (2023) 11:297. doi: 10.3390/healthcare11030297, 36766873 PMC9914781

[11] Taheri M Takian A Taghizadeh Z Jafari N Sarafraz N. Creating a positive perception of childbirth experience: systematic review and Meta-analysis of prenatal and intrapartum interventions. Reprod Health. (2018) 15:73. doi: 10.1186/s12978-018-0511-x, 29720201 PMC5932889

[12] Takegata M Haruna M Morikawa M Yonezawa K Komada M Severinsson E. Qualitative exploration of fear of childbirth and preferences for mode of birth among Japanese Primiparas. Nurs Health Sci. (2018) 20:338–45. doi: 10.1111/nhs.12571, 30311412

[13] Phunyammalee M Buayaem T Boriboonhirunsarn D. Fear of childbirth and associated factors among Low-risk pregnant women. J Obstet Gynaecol. (2019) 39:763–7. doi: 10.1080/01443615.2019.1584885, 31007101

[14] Richens Y Smith DM Lavender DT. Fear of birth in clinical practice: a structured review of current measurement tools. Sex Reprod Healthc. (2018) 16:98–112. doi: 10.1016/j.srhc.2018.02.010, 29804785

[15] Mudgal S Shafqat N. Tools for assessing childbirth fear: a comprehensive review and psychometric evaluation. WHO South East Asia J Public Health. (2024) 13:16–23. doi: 10.4103/who-seajph.Who-seajph_151_23, 39167131

[16] Wijma K Wijma B Zar M. Psychometric aspects of the W-Deq; a new questionnaire for the measurement of fear of childbirth. J Psychosom Obstet Gynaecol. (1998) 19:84–97. doi: 10.3109/01674829809048501, 9638601

[17] Lowe NK. Self-efficacy for labor and childbirth fears in nulliparous pregnant women. J Psychosom Obstet Gynaecol. (2000) 21:219–24. doi: 10.3109/01674820009085591, 11191169

[18] Zhang Q McAra-Couper J Lou Y Guo S Qiu P. Validation of the Chinese version of the fear of birth scale among pregnant women. Midwifery. (2024) 133:103986. doi: 10.1016/j.midw.2024.103986, 38642425

[19] Roosevelt L Low LK. Exploring fear of childbirth in the United States through a qualitative assessment of the Wijma delivery expectancy questionnaire. J Obstet Gynecol Neonatal Nurs. (2016) 45:28–38. doi: 10.1016/j.jogn.2015.10.005, 26815796

[20] Memon MA Ting H Cheah J-H Thurasamy R Chuah F Cham TH. Sample size for survey research: review and recommendations. J Appl Struct Equ Model. (2020) 4:i–xx. doi: 10.47263/JASEM.4(2)01

[21] Bujang MA Omar ED Baharum NA. A review on sample size determination for Cronbach's alpha test: a simple guide for researchers. Malays J Med Sci. (2018) 25:85–99. doi: 10.21315/mjms2018.25.6.9, 30914882 PMC6422571

[22] Marzi G Balzano M Marchiori D. K-alpha calculator-Krippendorff's alpha calculator: a user-friendly tool for computing Krippendorff's alpha inter-rater reliability coefficient. MethodsX. (2024) 12:102545. doi: 10.1016/j.mex.2023.102545, 39669968 PMC11636850

[23] Polit DF Beck CT. The content validity index: are you sure you know what's being reported? Critique and recommendations. Res Nurs Health. (2006) 29:489–97. doi: 10.1002/nur.20147, 16977646

[24] Watkins MW. Exploratory factor analysis: a guide to best practice. J Black Psychol. (2018) 44:219–46. doi: 10.1177/0095798418771807

[25] Boateng GO Neilands TB Frongillo EA Melgar-Quiñonez HR Young SL. Best practices for developing and validating scales for health, social, and behavioral research: a primer. Front Public Health. (2018) 6:149. doi: 10.3389/fpubh.2018.00149, 29942800 PMC6004510

[26] Wu J Hong Y Guan H Huang M Liang J Wen Z . Cross-cultural adaptation and psychometric evaluation of the Chinese version of childbirth experience questionnaire 2.0. Front Psych. (2025) 16:1488966. doi: 10.3389/fpsyt.2025.1488966, 40621561 PMC12226488

[27] Johnson R Slade P. Does fear of childbirth during pregnancy predict emergency caesarean section? BJOG. (2002) 109:1213–21. doi: 10.1046/j.1471-0528.2002.01351.x, 12452457

[28] Fenwick J Gamble J Nathan E Bayes S Hauck Y. Pre- and postpartum levels of childbirth fear and the relationship to birth outcomes in a cohort of Australian women. J Clin Nurs. (2009) 18:667–77. doi: 10.1111/j.1365-2702.2008.02568.x, 19239535

[29] Tanglakmankhong K Perrin NA Lowe NK. Childbirth self-efficacy inventory and childbirth attitudes questionnaire: psychometric properties of Thai language versions. J Adv Nurs. (2011) 67:193–203. doi: 10.1111/j.1365-2648.2010.05479.x, 21158905

[30] Gourounti K Kouklaki E Lykeridou K. Validation of the childbirth attitudes questionnaire in Greek and psychosocial characteristics of pregnant women with fear of childbirth. Women Birth. (2015) 28:e44–51. doi: 10.1016/j.wombi.2015.02.004, 25747734

[31] Wei J Liu J Zhang L Wu Y Fu C. Reliability and validity of the Chinese version of childbirth attitudes questionnaire. Chin. J. Nurs. (2016) 31:81–3. doi: 10.3761/j.issn.0254-1769.2016.05.019

[32] Chen C Hussein SZB Nasri NWM Yao J Qin Y Zhao Z . Fear of childbirth among pregnant women: a concept analysis. J Adv Nurs. (2024) 80:4476–87. doi: 10.1111/jan.16218, 38738562

[33] Sheen K Slade P. Examining the content and moderators of women's fears for giving birth: a Meta-synthesis. J Clin Nurs. (2018) 27:2523–35. doi: 10.1111/jocn.14219, 29243289

[34] Fenwick J Toohill J Creedy DK Smith J Gamble J. Sources, responses and moderators of childbirth fear in Australian women: a qualitative investigation. Midwifery. (2015) 31:239–46. doi: 10.1016/j.midw.2014.09.003, 25440298

[35] Yoosefi Lebni J Khalajabadi Farahani F Solhi M Ebadi Fard Azar F. Causes and grounds of childbirth fear and coping strategies used by Kurdish adolescent pregnant women in Iran: a qualitative study. J Reprod Infertil. (2021) 22:47–56. doi: 10.18502/jri.v22i1.4995, 33680885 PMC7903670

[36] Melender HL. Experiences of fears associated with pregnancy and childbirth: a study of 329 pregnant women. Birth. (2002) 29:101–11. doi: 10.1046/j.1523-536x.2002.00170.x, 12051188

[37] Hassan SJ Sundby J Husseini A Bjertness E. The paradox of vaginal examination practice during normal childbirth: Palestinian women's feelings, opinions, knowledge and experiences. Reprod Health. (2012) 9:16. doi: 10.1186/1742-4755-9-16, 22929060 PMC3560273

[38] Kululanga LI Malata A Chirwa E Sundby J. Malawian fathers’ views and experiences of attending the birth of their children: a qualitative study. BMC Pregnancy Childbirth. (2012) 12:141. doi: 10.1186/1471-2393-12-141, 23216825 PMC3520855

[39] Nori W Kassim MAK Helmi ZR Pantazi AC Brezeanu D Brezeanu AM . Non-pharmacological pain Management in Labor: a systematic review. J Clin Med. (2023) 12:7203. doi: 10.3390/jcm12237203, 38068274 PMC10707619

[40] Emelonye AU Pitkäaho T Aregbesola A Vehviläinen-Julkunen K. Spouses' perspective of their participation and role in childbirth pain relief. Ann Med Health Sci Res. (2016) 6:367–74. doi: 10.4103/amhsr.amhsr_12_16, 28540105 PMC5423337

[41] Serçekuş P Okumuş H. Fears associated with childbirth among nulliparous women in Turkey. Midwifery. (2009) 25:155–62. doi: 10.1016/j.midw.2007.02.005, 17600599

[42] Fisher C Hauck Y Fenwick J. How social context impacts on women's fears of childbirth: a Western Australian example. Soc Sci Med. (2006) 63:64–75. doi: 10.1016/j.socscimed.2005.11.065, 16476516

[43] Slade P Balling K Sheen K Houghton G. Establishing a valid construct of fear of childbirth: findings from in-depth interviews with women and midwives. BMC Pregnancy Childbirth. (2019) 19:96. doi: 10.1186/s12884-019-2241-7, 30885153 PMC6423809

[44] Eriksson C Westman G Hamberg K. Experiential factors associated with childbirth-related fear in Swedish women and men: a population based study. J Psychosom Obstet Gynaecol. (2005) 26:63–72. doi: 10.1080/01674820400023275, 15962723

[45] Wigert H Nilsson C Dencker A Begley C Jangsten E Sparud-Lundin C . Women's experiences of fear of childbirth: a Metasynthesis of qualitative studies. Int J Qual Stud Health Well-being. (2020) 15:1704484. doi: 10.1080/17482631.2019.1704484, 31858891 PMC6968519

[46] Lyberg A Severinsson E. Fear of childbirth: mothers' experiences of team-midwifery care - a follow-up study. J Nurs Manag. (2010) 18:383–90. doi: 10.1111/j.1365-2834.2010.01103.x, 20609042

[47] Salomonsson B Berterö C Alehagen S. Self-efficacy in pregnant women with severe fear of childbirth. J Obstet Gynecol Neonatal Nurs. (2013) 42:191–202. doi: 10.1111/1552-6909.12024, 23488555

[48] Huang J Huang J Li Y Liao B. The prevalence and predictors of fear of childbirth among pregnant Chinese women: a hierarchical regression analysis. BMC Pregnancy Childbirth. (2021) 21:643. doi: 10.1186/s12884-021-04123-7, 34551755 PMC8456556

[49] Olza I Leahy-Warren P Benyamini Y Kazmierczak M Karlsdottir SI Spyridou A . Women's psychological experiences of physiological childbirth: a Meta-synthesis. BMJ Open. (2018) 8:e020347. doi: 10.1136/bmjopen-2017-020347, 30341110 PMC6196808

